# Supplementary material for: Grounded Yet Ascending: Faculty Development in a Time of Reduced Budgets and Travel
Source: MedEdPublish (2016). 2026 Jun 23;16:24. [Version 1] doi: 10.12688/mep.21621.1 (PMC13554763; doi:10.12688/mep.21621.1)
Supplement: Peer review history

# Reviewer report: Reviewer response for version 1

**DOI:** 10.21956/mep.23166.r46104

**Published:** 2026-07-15

**Authors:** Fahad Azam, Abida Shaheen, Nourhan F. Wasfy

**Affiliations:** 1 Faculty of Health Sciences, Shifa Tameer-e-Millat University, Islamabad, Pakistan; 2 Suez University Faculty of Medicine (Ringgold ID: 619931), Suez, Suez Governorate, Egypt

This brief report explores the impact of recent changes in funding on faculty professional development opportunities at academic medical centers, particularly with respect to travel for conferences and professional meetings.

 The study has academic merit because it addresses an important challenge facing faculty development within academic medicine.

 The manuscript is well organized and clearly written, with a logical progression from the introduction through the discussion. The tables effectively summarize the survey findings and improve readability. The literature cited is relevant and current. However, the background could be strengthened by including a few additional references on faculty development frameworks and the educational and career value of conference participation.

 As with most exploratory surveys using voluntary participation, the findings should be interpreted within the context of the sampling approach. It would be helpful if the discussion more explicitly acknowledged that the convenience sample may limit the broader applicability and generalizability of the findings, particularly since most respondents represented institutions in the US. The manuscript would also be strengthened by acknowledging additional limitations, such as the predominance of respondents from US institutions and the limited sample size.

 The manuscript would benefit from including a brief description of how the survey was developed, expanding the description of the qualitative analysis used for the open-ended responses, and clarifying whether all survey responses were complete and, if applicable, how missing data were handled. These additions would enhance methodological transparency without requiring substantial revision.

 The practical recommendations offered by the authors are reasonable and likely to be useful for faculty members navigating reduced travel support. The manuscript would be further strengthened by ensuring that statements regarding the impact of funding reductions are framed within the context of this exploratory sample, avoiding implications of broad generalizability.

Have any limitations of the research been acknowledged?

Yes

Is the study design appropriate and does the work have academic merit?

Partly

Is the work clearly and accurately presented and does it cite the current literature?

Yes

If applicable, is the statistical analysis and its interpretation appropriate?

Yes

Are all the source data underlying the results available to ensure full reproducibility?

Yes

Are the conclusions drawn adequately supported by the results?

Yes

Are sufficient details of methods and analysis provided to allow replication by others?

Partly

Reviewer Expertise:

HPE, Bioethics, Quality Assurance, Accreditation, Pharmacogenetics, Pharmacogenomics, Leadership.

We confirm that we have read this submission and believe that we have an appropriate level of expertise to confirm that it is of an acceptable scientific standard.

# Reviewer report: Reviewer response for version 1

**DOI:** 10.21956/mep.23166.r46107

**Published:** 2026-07-02

**Authors:** Shari Whicker

**Affiliations:** 1 Carilion Clinic/ Virginia Tech Carilion School of Medicine, Roanoke, VA, USA

This brief report discusses the concept of funding cuts impacting travel support for medical faculty professional development, limiting conference attendance, networking, and career advancement. It recommends proactive planning, submitting for scholarship opportunities for highly-valued conferences, leveraging virtual and local opportunities, joining professional communities, seeking grants, and maximizing institutional resources to offset these challenges.

 I think readers will value this brief report and it will help them to be thoughtful about professional development planning. Particularly helpful is the reminder that external professional development opportunities are multi-faceted in their benefits to include opportunities for: networking, sharing scholarship, and participation in national leadership roles which are all critical elements for faculty growth. I think the term "non-essential" (fully recognizing this is a formal term used for budgeting and other purposes) might be left to interpretation in the eye of the beholder.

Have any limitations of the research been acknowledged?

Yes

Is the study design appropriate and does the work have academic merit?

Yes

Is the work clearly and accurately presented and does it cite the current literature?

Yes

If applicable, is the statistical analysis and its interpretation appropriate?

Not applicable

Are all the source data underlying the results available to ensure full reproducibility?

Yes

Are the conclusions drawn adequately supported by the results?

Yes

Are sufficient details of methods and analysis provided to allow replication by others?

Yes

Reviewer Expertise:

faculty development, rofessional development, communities of practice, teaching academies

I confirm that I have read this submission and believe that I have an appropriate level of expertise to confirm that it is of an acceptable scientific standard.
